# Isolated Subclavian Vein Injury: A Rare and High Mortality Case

**DOI:** 10.1155/2013/152762

**Published:** 2013-05-23

**Authors:** Sahin Iscan, Mustafa Etli, Ozgur Gursu, Esra Eker, Helin El Kilic

**Affiliations:** ^1^Department of Cardiovascular Surgery, Van Research and Education Hospital, Van 65100, Turkey; ^2^Department of Anesthesiology and Reanimation, Van Research and Education Hospital, Van 65100, Turkey

## Abstract

Isolated subclavian vein injuries are rarely seen without concomitant arterial injury, bone fracture, damage to brachial plexus, and thoracal traumas. Our case was brought to the emergency service 6 hours after he had been shot at the shoulder with a firearm. After detection of extravasation from the left axillary and subclavian vein on arteriographic and venographic examinations, he was operated on. An autogenous saphenous vein graft was interposed between subclavian and axillary veins. Cardiac arrest developed twice because of hypovolemia, which was resolved with medical therapy. Subclavian vein injuries have a more mortal course when compared with the injuries to the subclavian arteries. Its most important reason is excessive blood loss and air embolism because of delayed arrival to hospital. As is the case in all vascular injuries, angiography is the most important diagnostic examination. If the general health state of the patient permits, arteriography and venography should be performed in patients potentially exposed to vascular injuries. In patients with extreme blood loss and deteriorated health state, direct surgical exploration of the injury site, containment of the bleeding, and venous repair are life-saving approaches.

## 1. Introduction

Many surgeons have not sufficient clinical experience in the management of vascular injuries involving subclavian and axillary regions due to their rarity [1]. These vascular injuries can be accompanied with bone fractures, brachial plexus, and chest injuries. Penetrating injuries are more frequently seen, which include isolated venous (44%), arterial (39%), and mixed vascular (17%) injuries. Most (61%) of these patients could not get a hospital care, and 15% of the operated surgical patients lose their lives. Contrary to expectations, venous injuries (VI) have a more mortal course relative to arterial injuries. Air embolism and excessive blood loss due to delayed referrals to hospital may be the basic reason [2, 3]. 

In this case report, surgical treatment of a patient, who was brought to hospital with excessive blood loss resulting from being shot at shoulder region with a firearm, with the indication of isolated subclavian vein injury is presented.

## 2. Case Presentation

**Figure 1:**
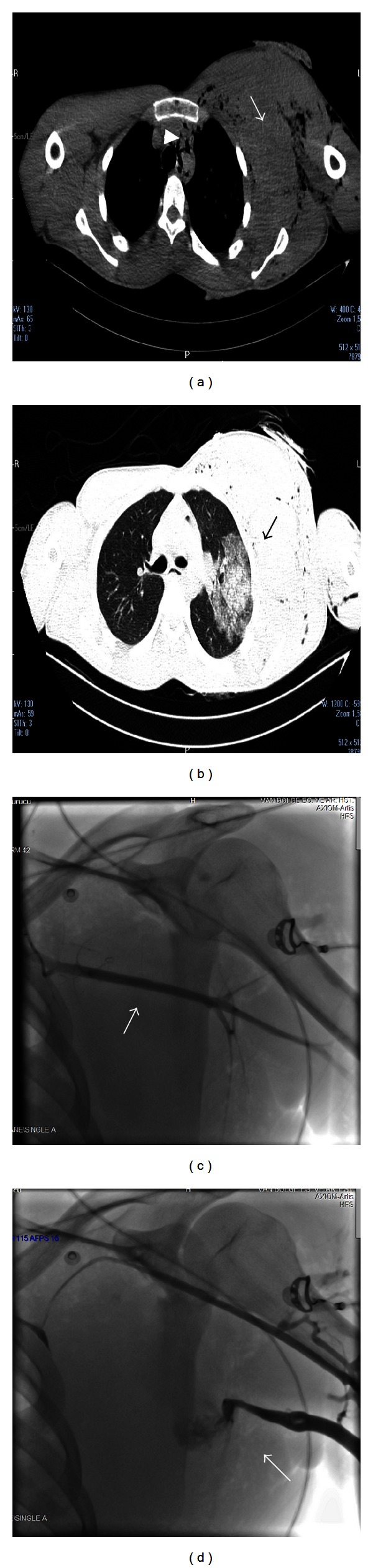
(a) Hematoma, subcutaneous emphysema (arrow) in the left hemithorax, and air in the mediastinum (arrowheads). (b) Pulmonary contusion (arrow). (c) Arteriogram showing patent subclavian artery (arrow). (d) Venogram demonstrating extravasation from axillary vein (arrow).

**Figure 2:**
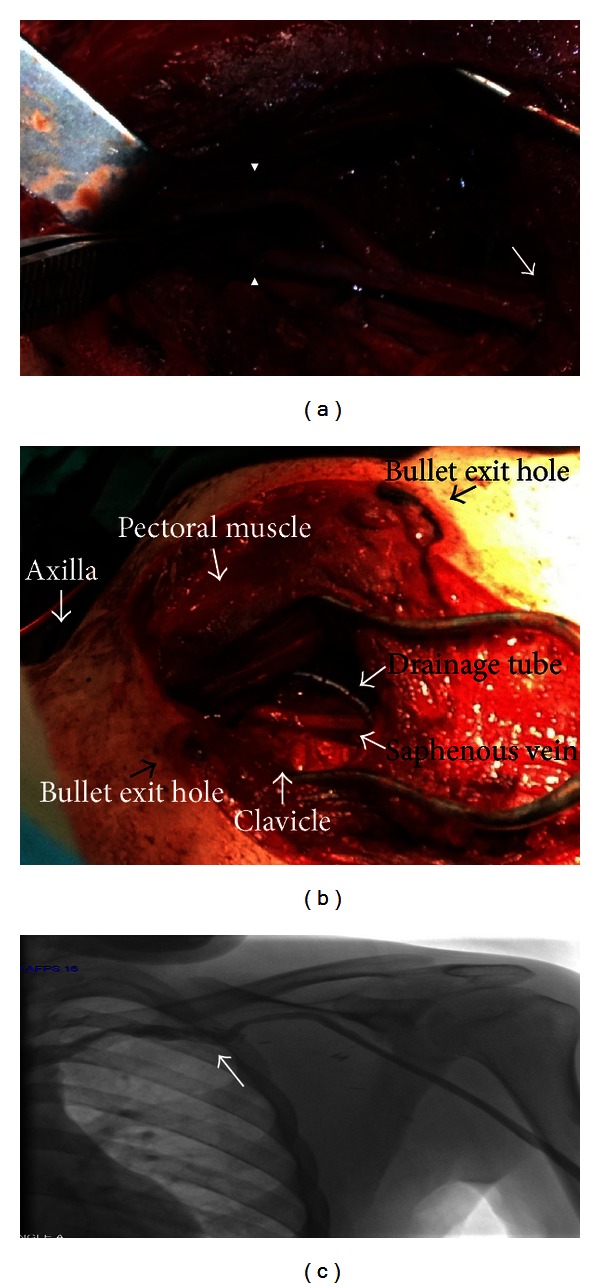
(a) Repaired venous segment; proximal (arrow) and distal anastomoses (arrowhead). (b) Injury site and incision line after vascular repair. (c) Venogram demonstrating venous blood flow obtain at postoperative 1st month.

A 20-year-old male patient was consulted to the emergency service, nearly 6 hours after a firearm injury directed at his left infraclavicular region. Arterial blood pressure was 100/60 mm Hg, and heart rate was 105/min. Presumed bullet entrance hole was located nearly 3 cm above the left scapula, and its exit hole was approximately 12 cm above the left nipple. On the anterior aspect of the left hemithorax, a marked swelling when compared with the right hemithorax, hematoma, and subcutaneous emphysema which caused respiratory distress were noted. Presumably venous blood was oozing from the exit hole on this site. Arterial pulses were palpable, and any sign of ischemia was not detected on the upper extremity. Radiograms obtained did not show any evidence of pneumothorax, hemothorax, or bone fracture. Computed tomograms revealed a widespread subcutaneous hematoma, emphysema covering all over the anterior aspect of the left hemithorax, diffuse contusion of the left lung, and mediastinal air. The patient was taken into the angiography unit. Selective left subclavian artery imaging demonstrated intact subclavian artery. Venographic examination disclosed extravasation in the subclavian vein extending from just proximal to the axillary vein (Figure 1). The patient was urgently operated with a severely decreased hematocrit (18%). Through a 5 cm long incision, axillary artery and vein were explored. Still through a nearly 15 cm long incision extending from one-third medial of the clavicula in the left infraclavicular region and encompassing the wounded area, pectoralis major muscle was partially excised, and on the medial side, subclavian artery and vein were explored. Distally, at the shoulder level a nearly 5 cm defect created by axillary vein rupture extending up to its insertion into the subclavian vein which roughly corresponds to the midpoint of clavicula was observed. Abundant venous bleeding was noted oozing through this site. Proximal and distal explorations revealed terminal ends of veins; then vascular clamps were placed, so as to stop bleeding. The patient was heparinized. Through a right femoral incision, saphenous vein was brought out and interposed between axillary and subclavian veins (Figure 2). Subclavian artery and brachial plexus were observedly intact. Muscular structures were repaired, and adequate debridement was ensured. A total of 2100 cc blood and blood product were replaced intraoperatively. At the start of the operation, before hemostatic control, and at the end of the operation, following anastomosis, cardiac arrest occurred and heart rhythm was corrected with medical therapy.

## 3. Discussion

Subclavian vein injuries are rarely seen, more frequently caused by firearms, and result in disability, extremity loss, and mostly death. VIs are generally detected intraoperatively by chance in patients operated for traumas [4]. Isolated VI may not present with diagnostic physical symptoms. Absence of any arterial injury, concomitant abnormality in the presence of only bleeding, and hematoma might divert the surgeon from the possibility of VI in case of inadequate evaluation.

Spontaneous cessation of bleeding in cases with VI is a rare possibility due to weakened venous contractility. Infraclavicular and retroclavicular locations of most injuries complicate exploration. In this case, because of diffuse venous bleeding coming out from bullet exit hole, exploration time was prolonged, and cardiac arrest developed secondary to hypovolemia. In studies performed, required amount of blood transfusion was found to be approximately 3550 mL for arterial, and 5125 mL for VI. This indicates more risky progression of venous injuries. In venous bleedings, as is in this case, incisions should be extended to the injury site, major pectoral muscles should be excised to ensure a ready exposure for hemostatic control. If required, clavicula can be excised. In the management of proximal subclavian vascular injuries, median sternotomy can be performed as well [1].

Angiography is the most important diagnostic tool especially in cases with vascular injuries. Some investigations have demonstrated that diagnostic arteriography delays diagnosis and treatment, and also clinical evaluation ranks first in priority over diagnostic arteriography [4, 5]. In this case, venography was performed in addition to arteriography. In settings where imaging techniques are not available, larger hematoma, subcutaneous emphysema, and hypovolemia should direct the surgeon to emergent surgical exploration.

Comparison of reparative methods and ligation for the management of VI demonstrated only mild edema formation after ligation [1]. In cases with concomitant arterial and VI, since larger tissue defect is the issue and venous collateral circulation will not be able to provide adequate perfusion, venous repair should be also performed if prolonged surgery will not jeopardize patient's life. In this case, despite one incident of cardiac arrest, thanks to the young age of the patient, venous repair was performed.

 In conclusion, isolated subclavian vein injuries are rarely seen. Contrary to expectations, mortality rates after subclavian vein injuries are much higher than those of the arterial injuries. If the general health state of the patient permits, arteriographic and venographic examinations should be performed in patients with presumed vascular injuries. In patients with excessive blood loss and deteriorated health state, direct exploration of the injury site, hemostatic control, and venous repair are life-saving interventions.

## References

[1] Demetriades D, Chahwan S, Gomez H (1999). Penetrating injuries to the subclavian and axillary vessels. *Journal of the American College of Surgeons*.

[2] Demetriades D, Rabinowitz B, Pezikis A, Franklin J, Palexas G (1987). Subclavian vascular injuries. *British Journal of Surgery*.

[3] Guloglu R, Bilsel Y, Alis H, Ertekin C, Kurtoglu M (1999). Traumatic subclavian and axillary vessel injuries. *International Journal of Angiology*.

[4] Graham JM, Mattox KL, Feliciano DV, DeBakey ME (1982). Vascular injuries of the axilla. *Annals of Surgery*.

[5] de Silva W, Ubayasiri R, Weerasinghe C, Wijeyaratne S (2011). Challenges in the management of extremity vascular injuries: a wartime experience from a tertiary centre in Sri Lanka. *World Journal of Emergency Surgery*.

